# Nonalcoholic fatty liver disease is a risk factor for large-for-gestational-age birthweight

**DOI:** 10.1371/journal.pone.0221400

**Published:** 2019-08-26

**Authors:** Seung Mi Lee, Byoung Jae Kim, Ja Nam Koo, Errol R. Norwitz, Ig Hwan Oh, Sun Min Kim, Sang Youn Kim, Gyoung Min Kim, Soo Heon Kwak, Won Kim, Sae Kyung Joo, Sue Shin, Chanthalakeo Vixa, Chan-Wook Park, Jong Kwan Jun, Joong Shin Park

**Affiliations:** 1 Department of Obstetrics and Gynecology, Seoul National University College of Medicine, Seoul, Korea; 2 Department of Obstetrics and Gynecology, Seoul Metropolitan Government Seoul National University Boramae Medical Center, Seoul, Korea; 3 Seoul Women's Hospital, Incheon, Korea; 4 Department of Obstetrics and Gynecology, Tufts University School of Medicine, Boston, Massachusetts, United States of America; 5 Department of Radiology, Seoul National University College of Medicine, Seoul, Korea; 6 Department of Radiology, Yeonsei University College of Medicine, Seoul, Korea; 7 Department of Internal Medicine, Seoul National University College of Medicine, Seoul, Korea; 8 Department of Internal Medicine, Seoul Metropolitan Government Seoul National University Boramae Medical Center, Seoul, Korea; 9 Department of Laboratory Medicine, Seoul National University College of Medicine, Seoul, Korea; 10 Department of Laboratory Medicine, Seoul Metropolitan Government Seoul National University Boramae Medical Center, Seoul, Korea; 11 Department of Obstetrics & Gynecology, University of Health Sciences, Vientiane, Lao PDR; National Institute of Public Health, MEXICO

## Abstract

**Objective:**

Nonalcoholic fatty liver disease (NAFLD) is a well-recognized hepatic manifestation of metabolic disease in adults and has been associated with the development of gestational diabetes (GDM). Hepatic insulin resistance can result in increased release of glucose (from gluconeogenesis) and free fatty acids (due to enhanced lipolysis), which can lead in turn to fetal overgrowth. However, the relationship between maternal metabolic factors (such as circulating levels of triglycerides, free fatty acids [FFA], or adipokines) and excessive fetal birthweight in NAFLD has not been carefully examined. In this study, we evaluated the relationship between NAFLD and the subsequent risk of large-for-gestational-age (LGA) birthweight.

**Method:**

Singleton nondiabetic pregnant women were evaluated for the presence of fatty liver at 10–14 weeks of gestation by abdominal ultrasound. The degree of fatty liver was classified as Grade 0–3 steatosis. At the time of liver ultrasound, maternal blood was taken after fasting and measured for adiponectin and FFA. LGA was defined as birthweight >90^th^ percentile for gestational age.

**Results:**

A total of 623 women were included in the analysis. The frequency of LGA was 10.9% (68/623), and the frequency of NAFLD was 18.9%. The risk of LGA increased significantly in patients with Grade 2–3 steatosis in the first trimester. The relationship between Grade 2–3 steatosis and LGA remained significant after adjustment for maternal age, pre-pregnancy BMI, GDM, and maternal serum triglyceride levels. The concentration of maternal blood adiponectin at 10–14 weeks was significantly lower in cases with LGA than non-LGA, but the maternal blood FFA concentrations were not different between the groups.

**Conclusion:**

The presence of Grade 2–3 steatosis on ultrasound in early pregnancy was associated with the increased risk of delivering an LGA infant, even after adjustment for multiple confounding factors including GDM. Adiponectin may be the linking biomarker between NAFLD and LGA.

## Introduction

Abnormal fetal growth—both fetal growth restriction and excessive fetal growth—is a risk factor for obesity and cardiovascular disease in later life.[1–3] Fetal growth is dependent on the adequate supply of nutrients across the placenta, but the exact mechanisms regulating fetal growth are not fully understood. Maternal hyperglycemia is one of the most important drivers of fetal growth, but excessive fetal weight gain also happens in euglycemic mothers.[4, 5]

Nonalcoholic fatty liver disease (NAFLD) is defined as an abnormal accumulation of fat within the liver in the absence of viral hepatitis, hepatobiliary disease, or excessive alcohol consumption, and is one of the most common liver diseases in adults.[6–9] NAFLD is a hepatic manifestation of metabolic syndrome, and is associated with type 2 diabetes.[10, 11] It has been suggested that NAFLD may be associated with gestational diabetes (GDM).[12, 13] To examine this relationship between NAFLD and GDM, we enrolled pregnant women in a prospective cohort study, the “Fatty liver in pregnancy” registry (NCT02276144), and reported that pregnant women diagnosed as NAFLD in early pregnancy were at high risk for GDM even after adjustment for metabolic risk factors[14].

Considering that the hepatic insulin resistance that accompanies pregnancy can result in increased release of glucose (from gluconeogenesis) and free fatty acids (due to enhanced lipolysis), it is plausible that NAFLD can lead to fetal overgrowth. However, the relationship between maternal metabolic factors (such as circulating levels of triglycerides, free fatty acids [FFA], or adipokines), NAFLD, and excessive fetal birthweight has not been carefully examined. In this study, we evaluate the relationship between NAFLD and the subsequent risk of large-for-gestational-age (LGA) birthweight.

## Materials and methods

### Study design

A prospective cohort study, the “Fatty liver in pregnancy” registry (NCT02276144), was performed at Incheon Seoul Women’s Hospital and Seoul Metropolitan Government Seoul National University Boramae Medical Center to determine the risk of NAFLD on pregnancy outcome[14]. Singleton pregnant women who visited outpatient clinic for prenatal check were offered to participate in the prospective cohort, if they were in the first trimester period (before 14 weeks of gestation). After informed consent, baseline clinical and demographic characteristics (including a history of obstetric or medical diseases such as liver disease or diabetes, family history of diabetes, pre-gestational weight and height) were asked by questionnaire at the time of enrollment. The study was approved by the Institutional Review Board of the Seoul Metropolitan Government Seoul National University Boramae Medical Center and by the Public Institutional Review Board of the Ministry of Health and Welfare of Korea. Written patient consent was obtained.

At 10–14 weeks of gestation, patients were evaluated for NAFLD using liver ultrasound. Patients were followed throughout the course of their pregnancy. Patients were excluded from the final analysis if they: (1) had evidence of preexisting chronic liver disease, excessive alcohol consumption, or pre-gestational diabetes; (2) were lost to follow-up; or (3) had a preterm birth before 34 weeks.

### Definition of LGA

LGA was defined as birthweight >90^th^ percentile for gestational age using data derived from a Korean population[15]. The information of birthweight were gathered from birth records in medical records. In our institution, newborns are routinely measured for birthweight at the time of delivery.

### Determination of NAFLD

Maternal liver ultrasound was performed at 10–14 weeks’ gestation by an experienced ultrasound examiner. Liver ultrasound was performed at the time of fetal ultrasound using E8 ultrasound machine (General Electronic, Austria), Accuvix XQ ultrasound machine (Samsung Medison, Seoul, Korea), or WS80A ultrasound machine (Samsung Medison, Seoul, Korea).

NAFLD was diagnosed by the presence of bright liver echo patterns in ultrasonography. The extent of NAFLD was classified according to established criteria:[16] (i) Grade 0 steatosis: normal echogenicity; (ii) Grade 1 steatosis: slight, diffuse increase in fine echoes in liver parenchyma with normal visualization of the diaphragm and intrahepatic vessel borders; (iii) Grade 2 steatosis: moderate, diffuse increase in fine echoes with slightly impaired visualization of intrahepatic vessels and the diaphragm; (iv) Grade 3 steatosis: marked increase in fine echoes with poor or no visualization of intrahepatic vessel borders, the diaphragm, and the posterior right lobe of the liver. Stored images were subsequently reviewed by 2 experienced radiologists (SYK, GMK) who were blinded to the clinical characteristics of the patients.

In addition to sonographic grading of NAFLD, a fatty liver index (FLI) was calculated for each subject using the following formula[17];

$$\text{FLI}=(\text{exp}[{\text{Model}}_{\text{FLI}}])/(1+\text{exp}[{\text{Model}}_{\text{FLI}}])\times 100$$

where Model_FLI_ = 0.953×ln (TG) + 0.139×BMI + 0.718×ln (GGT) + 0.053×WC − 15.745.

The FLI was classified into 3 categories[18]: (1) Low-risk group for fatty liver, FLI ≤20; (2) Intermediate-risk group, 20 < FLI < 60; and (3) High-risk group, FLI ≥60.

### Measurement of free fatty acid and adiponectin

At the time of liver ultrasound (10–14 weeks), a venous blood sample was collected after fasting at least 8 hours, centrifuged, aliquoted, and stored at -70°C for subsequent analysis. Stored blood samples were thawed, batched, and measured for FFA using coupled enzymatic reaction system (ACS-ACOD Method, Roche, Germany) and for adiponectin using enzyme-linked immunosorbent assay (R&D systems, United States).

### Statistical analysis

Proportions were compared with Fisher’s exact test or chi-square test as appropriate; continuous variables were compared with the Mann-Whitney U test. Logistic regression was performed to determine the relationship between NAFLD and LGA after adjustment for confounding variables.

A prior sample size calculation was performed to determine how many women would be needed in the current study. We estimated the frequency of LGA and Grade 2–3 steatosis in the pregnant women would be 10% and 5%, respectively, according to the previous report[19]. With 80% power and a type 1 error of 5%, we determined that we would require 701 women if the frequency of LGA is increased to 30% in the presence of Grade 2–3 steatosis, and would require 361 women if the frequency of LGA is increased to 40% in the presence of Grade 2–3 steatosis.

A p-value of <0.05 was considered statistically significant. SPSS version 21.0 software (SPSS Inc., Chicago, IL) was utilized.

## Results

Between November 2014 and July 2016, a total of 663 women without chronic liver disease, excessive alcohol use, or pre-gestational diabetes underwent liver ultrasound at 10–14 weeks of gestation. After excluding women who were lost to follow-up (n = 35) and those who delivered before 34 weeks (n = 5), 623 women were included in the final analysis. Among these, the frequency of NAFLD diagnosed by liver ultrasound was 18.9% (Grade 1 steatosis 14.4% [90/623] and Grade 2–3 steatosis 4.5% [28/623]). FLI was calculated in 599 (96.1%) of the 623 study subjects. Of these, 424 (70.8%) were in the low-risk FLI group; 143 (23.9%) were intermediate-risk, and 32 (5.3%) were high-risk.

Table 1 shows the clinical characteristics of women who did not (Group 1) and who did (Group 2) deliver an LGA neonate. In this cohort, 10.9% (68/623) of women delivered LGA neonates (Group 2). These women had a higher pre-pregnancy BMI and were more likely to develop GDM. In the laboratory tests performed at the time of liver ultrasound, Women who delivered LGA neonates had higher level of triglyceride and homeostasis model assessment-insulin resistance (HOMA-IR) than those who did not.

**Table 1:** Characteristics, laboratory results, and pregnancy outcomes of study population.

Characteristics	Group 1 Women who delivered non-LGA neonates (n = 555)	Group 2 Women who delivered LGA neonates (n = 68)	p-value
Baseline characteristics			
Age (years)	32 (30–34)	32 (29–35)	NS
Nulliparity	285 (51%)	39 (57%)	NS
Pre-pregnancy BMI (kg/m^2^)	21.3 (19.5–23.5)	23.0 (20.5–25.2) (n = 67)	<0.005
Pre-pregnancy BMI ≥25 kg/m^2^	87 (16%)	17/67 (25%)	<0.05
Laboratory result at 10–14 weeks	(n = 538)	(n = 65)	
- AST (IU/L)	16 (14–20) (n = 537)	17 (15–21) (n = 64)	0.180
- ALT (IU/L)	11 (8–15) (n = 537)	12 (9–19) (n = 64)	0.143
- Cholesterol (mg/dL)	171 (154–190)	170 (152–192)	NS
- HDL-cholesterol (mg/dL)	65 (56–74)	64 (50–73)	NS
- LDL-cholesterol (mg/dL)	84 (70–98)	83 (70–97)	NS
- Triglyceride (mg/dL)	110 (86–139)	119 (95–154)	<0.05
- GGT (IU/L)	12 (10–15)	12 (10–18)	NS
- Fasting glucose (mg/dL)	77 (71–83) (n = 537)	79 (70–85)	0.154
- Insulin (μIU/mL)	8.2 (5.4–11.5) (n = 537)	10.4 (7.0–16.8)	<0.005
- HOMA-IR	1.5 (1.0–2.3) (n = 537)	2.0 (1.2–3.2)	<0.005
Pregnancy outcomes	(n = 555)	(n = 68)	
Gestational diabetes	30/543 (6%)	9/67 (13%)	<0.05
Gestational hypertension or preeclampsia	15/546 (3%)	2/66 (3%)	NS
Gestational age at delivery (weeks)	39.1 (38.3–40.0)	39.2 (38.0–40.1)	NS
Cesarean delivery	198 (36%)	30 (44%)	NS
Birthweight (kg)	3.18 (2.94–3.38)	3.86 (3.73–4.07)	<0.001
Infant gender (male)	275 (50%)	40 (59%)	NS

Data are presented as median and interquartile range or n (%)

BMI, body mass index; LGA, large-for-gestational age; AST, aspartate aminotransferase; ALT, alanine aminotransferase; GGT, gamma-glutamyl transferase; and HOMA-IR, homeostasis model assessment-insulin resistance.

Table 2 compares the results of liver ultrasound and laboratory tests between the two groups. Women who delivered LGA neonates had a higher proportion of Grade 2–3 steatosis and intermediate-/high-risk FLI than those who did not.

**Table 2:** Liver ultrasound and laboratory results at 10–14 weeks of gestation.

Characteristics	Group 1, Women who delivered non-LGA neonates(n = 555)	Group 2, Women who delivered LGA neonates(n = 68)	p-value
Gestational age at liver ultrasound (weeks)	12.4 (12.1–12.9)	12.4 (12.0–12.6)	NS
Fatty liver on liver ultrasound			<0.005
- Grade 0 steatosis (normal)	454 (82%)	51 (75%)	
- Grade 1 steatosis	82 (15%)	8 (12%)	
- Grade 2–3 steatosis	19 (3%)	9 (13%)	
Presence of Grade 2/3 fatty liver on liver ultrasound	19 (3%)	9 (13%)	<0.005
Fatty liver index (FLI)			<0.005
- Low-risk (FLI≤20)	391/535 (73%)	33/64 (52%)	
- Intermediate-risk (20<FLI<60)	120/535 (22%)	23/64 (36%)	
- High-risk (FLI≥60)	24/535 (5%)	8/64 (13%)	
Free fatty acid at liver ultrasound (μEq/L)	607 (462–784) (n = 537)	672 (445–869) (n = 65)	NS
Adiponectin at liver ultrasound (ng/mL)	5148 (2995–8194) (n = 538)	4116 (1814–7193) (n = 65)	<0.05

Data are presented as median and interquartile range or n (%).

The frequency of delivering an LGA neonate increased significantly in patients with Grade 2–3 steatosis in the first trimester: 10% in Grade 0 steatosis vs. 9% in Grade 1 steatosis vs. 32% in Grade 2–3 steatosis (p<0.005). The frequency of LGA neonates also increased significantly according to the FLI: 8% in low-risk, 16% in intermediate-risk, and 25% in high-risk (p<0.005). The relationship between Grade 2–3 steatosis and LGA remained significant after adjustment for maternal age (Model 1), for maternal age and GDM (Model 2), for maternal age, GDM, pre-gestational BMI, and triglyceride level (Model 3), and even for maternal age, GDM, pre-gestational BMI, triglyceride level, and HOMA-IR (Model 4) (Table 3). This same relationship exists also between FLI and LGA and remained significant after multiple adjustments (Table 3).

**Table 3:** Relationship of various independent variables with the risk of large-for-gestational age analyzed by multiple logistic regression analysis.

Characteristics	Grade 2–3 steatosis	p-value
Unadjusted (n = 623)	4.303 (1.863–9.942)	<0.005
Model 1 (n = 623)	4.274 (1.848–9.884)	<0.005
Model 2 (n = 610)	3.601 (1.418–9.142)	<0.01
Model 3 (n = 589)	3.164 (1.158–8.643)	<0.05
Model 4 (n = 588)	3.157 (1.160–8.594)	<0.005
	Intermediate-/high-risk fatty liver index	
Unadjusted (n = 599)	2.551 (1.507–4.317)	<0.001
Model 1 (n = 599)	2.584 (1.524–4.380)	<0.005
Model 2 (n = 586)	2.248 (1.278–3.954)	<0.01
Model 3 (n = 586)	2.331 (1.115–4.871)	<0.05
Model 4 (n = 585)	2.156 (1.025–4.538)	<0.05

Model 1: Adjusted for maternal age

Model 2: Adjusted for maternal age + gestational diabetes

Model 3: Adjusted for maternal age + gestational diabetes + Pre-pregnancy BMI + triglyceride level

Model 4: Adjusted for maternal age + gestational diabetes + Pre-pregnancy BMI + triglyceride level + HOMA-IR

**Fig 1:**
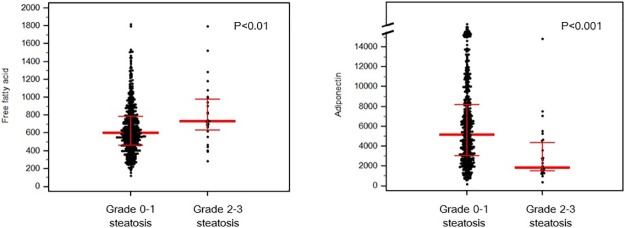
Maternal blood free fatty acid and adiponectin concentrations according to the grade of fatty liver at 10–14 weeks of gestation. (a) Free fatty acid (μEq/L). (b) Adiponectin (ng/mL).

**Fig 2:**
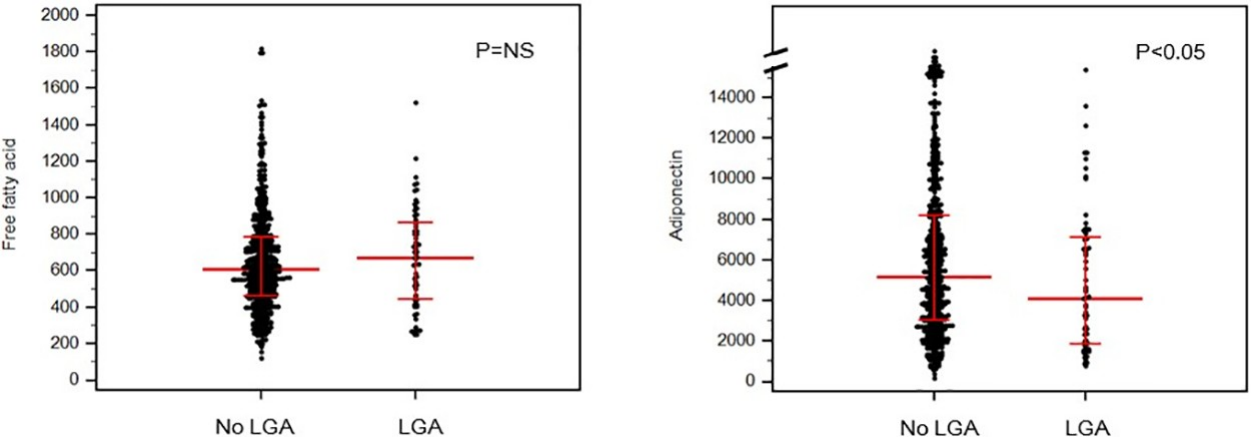
Maternal blood free fatty acid and adiponectin concentrations according to birthweight at the time of delivery. (a) Free fatty acid (μEq/L). (b) Adiponectin (ng/mL).

In maternal blood taken at the time of liver ultrasound (10–14 weeks), the mean concentration of FFA was significantly higher and that of adiponectin was significantly lower in patients with Grade 2–3 steatosis than those without Grade 2–3 steatosis (Fig 1). In addition, FFA concentrations were not different between women who did and did not deliver LGA neonates, whereas adiponectin levels were significantly lower in women who delivered LGA neonates compared to those who did not deliver an LGA neonate (Fig 2).

## Discussion

The principal findings of this study were: (1) in this cohort, the frequency of LGA was 10.9% (68/623) and of NAFLD was 18.9%; (2) the risk of LGA increased significantly in patients with Grade 2–3 steatosis in the first trimester; (3) the relationship between Grade 2–3 steatosis and LGA remained significant after adjustment for age, pre-gestational BMI, GDM, maternal serum triglyceride levels, and HOMA-IR; and (4) levels of adiponectin in the maternal circulation were significantly lower in women who delivered an LGA neonate, even at the time of 10–14 weeks of gestation.

In the current study, we demonstrated an association between NAFLD (defined both by ultrasound and using a validated biochemical index, the FLI) and LGA. In the one prior publication investigating this association, fatty liver was diagnosed using an elevated serum alanine aminotransferase in the first trimester,[20] which is not a sufficient marker of NAFLD. In this study, the ALT ≥90th percentile was associated with a 4-fold increased odds ratio of LGA. In the current study, we used far more rigorous criteria to diagnose NAFLD and investigated the association after adjustment for multiple co-variants, including amongst others GDM, BMI, and HOMA-IR.

In the current study, we demonstrated that NAFLD, not only diagnosed by ultrasound but also suggested by fatty liver index, was associated with LGA. As the interpretation of ultrasound may be dependent on examiner, we also evaluated the association between fatty liver index and LGA. However, the usefulness of fatty liver index should be validated in pregnant women.

In several previous studies, maternal triglyceride levels have been associated with excessive fetal growth, especially in non-diabetic pregnant women.[21–24] In the current study, mean maternal triglyceride levels were significantly higher at 10–14 weeks of gestation in women who subsequently delivered an LGA neonate (Table 2). However, this association did not remain significant after adjustment for NAFLD. Since circulating triglyceride levels may be a surrogate marker for NAFLD, the association between triglyceride and LGA is most likely due to the association between NAFLD and LGA.

In an effort to identify factors that may mediate the association between NAFLD and LGA, we tested FFA and adiponectin, both of which are potential biomarkers for fetal growth.[21, 25, 26] In the literature, the role of FFA in fetal growth is controversial, as FFA levels appear to be related to neonatal weight in patients with well controlled GDM, but not in nondiabetic pregnancies. [27]. In the current study, circulating levels of FFA in the first trimester were not associated with subsequent risk of LGA, although FFA levels were elevated in women with Grade 2–3 steatosis. This finding suggests that FFA, at least in the first trimester, is not a determining factor for fetal growth. In contrast, maternal blood adiponectin in the first trimester was associated with both NAFLD and LGA, suggesting that adiponectin may be functionally important in mediating this association. The association between maternal adiponectin levels and fetal growth is well documented.[28] Here, we showed that maternal adiponectin levels were associated with NAFLD as well as fetal growth. Whether this is true also of other adipokines such as leptin remains to be determined.

A major strength of this study is that we prospectively enrolled patients for determination of NAFLD and collected clinical information and maternal fasting blood in the first trimester. We also determined the presence of NAFLD using two well-validated approaches, namely liver ultrasound and biochemical testing (FLI). To our knowledge, this is the first study to demonstrate an association between NAFLD and LGA. Moreover, the association between NAFLD and adiponectin and the subsequent risk for LGA was evident already in the first trimester, suggesting increased risk of LGA at early stages of pregnancy. Prediction of LGA in the first trimester may allow for early interventions to mitigate this risk, such as modification of weight gain or physical activity during pregnancy. This information might to be relevant for clinicians, because they can aware and monitor fetal growth in these women. However, before change in clinical conducts or in guidelines, more studies are needed to confirm the result of the current study, as the current study is an observational study.

Then why is NAFLD associated with the risk of LGA? Fetal growth is determined by maternal provision of substrate and placental transfer of these substrates, but the precise cellular or hormonal mechanism has not been well understood. Several hormones are suggested to modulate these mechanisms, such as insulin, insulin-like growth factors, and adipokines. NAFLD is known as hepatic manifestation of metabolic syndrome, hepatic insulin resistance may impact fetal birthweight. However, the current study suggests that the relationship between NAFLD and LGA is independent of insulin resistance (HOMA-IR). In addition, adipokines such as adiponectin may modulate the relationship between NAFLD and LGA. In the current study, adiponectin was decreased in both NAFLD and LGA. Other adipokines such as leptin would clarify the role of adipokines in NAFLD and fetal growth.

Fetal growth is also dependent on maternal supply of nutrients. First, maternal glucose availability is one of the major determining factors, and there have been strong evidences between maternal hyperglycemia and fetal overgrowth [29]. Indeed, the frequency of gestational diabetes was also increased in patients with LGA in the current study. But the risk of LGA was associated with NAFLD, even after adjustment for GDM. Increased transfer of lipids can also result in fetal overgrowth, and Yarrington et al suggested that NAFLD may represent increased visceral adipose tissue, resulting increased FFA and fetal overgrowth[20]. However, we measured the FFA concentrations in the study population, and found that FFA was not related with the risk of LGA.

## Conclusion

The presence of Grade 2–3 steatosis on ultrasound in early pregnancy was associated with the increased risk of delivering an LGA infant, even after adjustment for GDM. Adiponectin may be the linking biomarker between NAFLD and LGA.

## Supporting information

S1 File This is the database file of the current manuscript. (SAV)
