# Correction: Nonalcoholic fatty liver disease is a risk factor for large-for-gestational-age birthweight

**DOI:** 10.1371/journal.pone.0224845

**Published:** 2019-10-30

**Authors:** Seung Mi Lee, Byoung Jae Kim, Ja Nam Koo, Errol R. Norwitz, Ig Hwan Oh, Sun Min Kim, Sang Youn Kim, Gyoung Min Kim, Soo Heon Kwak, Won Kim, Sae Kyung Joo, Sue Shin, Chanthalakeo Vixay, Chan-Wook Park, Jong Kwan Jun, Joong Shin Park

The thirteenth author’s name is spelled incorrectly. The correct name is: Chanthalakeo Vixay.
